# Glycated albumin and composite glycemic-lipid indices: Complementary tools for type 2 diabetes diagnosis

**DOI:** 10.7555/JBR.39.20250322

**Published:** 2026-09-28

**Authors:** Vera Lukić, Ana Ninić, Gordana Džingalašević, Slađana Menković, Ana Ružanović, Neda Milinković

**Affiliations:** 1 Department of Laboratory Diagnostics, Railway Healthcare Institute, 11000 Belgrade, Serbia; 2 Department of Medical Biochemistry, University of Belgrade, Faculty of Pharmacy, 11000 Belgrade, Serbia; 3 Department of Laboratory Diagnostics, Healthcare Institute of the Ministry of Internal Affairs, 11000 Belgrade, Serbia

**Keywords:** glycated albumin, type 2 diabetes mellitus, HDL-cholesterol, composite glycemic-lipid indices

## Abstract

Glycated albumin (GA) is a promising glycemic biomarker that offers advantages over glycated hemoglobin (HbA1c), especially under conditions that affect red blood cell turnover. In type 2 diabetes (T2DM), disturbances in glucose and lipid metabolism often coexist. This study aimed to evaluate the discriminatory performance of GA, HbA1c, and their composite indices incorporating high-density lipoprotein cholesterol (HDL-C) in a Serbian cohort of participants with established T2DM and controls. A total of 124 adults (including both T2DM patients and healthy controls) were analyzed for GA, HbA1c, glucose, and lipid profiles. Composite indices, namely, GA/HDL-C and HbA1c/HDL-C, were calculated. Logistic regression and receiver operating characteristic (ROC) analyses were used to assess discriminatory performance. GA and HbA1c levels were significantly higher in T2DM patients, while HDL-C levels were lower. Both GA/HDL-C and HbA1c/HDL-C showed strong associations with T2DM. HbA1c exhibited the highest area under the ROC curve (0.966), followed by HbA1c/HDL-C (0.883), GA/HDL-C (0.859), and GA (0.855). The GA/HDL-C index exhibited particularly high specificity (92.06%). GA levels in the control group were consistent with reference values reported in other populations. To our knowledge, this is the first study to report GA data in a Serbian cohort. The findings highlight GA as a useful complementary marker for distinguishing individuals with established T2DM from controls. Composite indices, particularly GA/HDL-C and HbA1c/HDL-C, may provide complementary information for integrated glycemic-lipid assessment.

## Introduction

Diabetes mellitus is one of the most prevalent chronic non-communicable diseases, currently affecting more than 537 million adults worldwide, with an equally large number of individuals at high risk of developing the disease because of impaired glucose tolerance^[1]^. This chronic metabolic disorder arises from insufficient insulin secretion and/or decreased tissue sensitivity to insulin, and is characterized by persistent hyperglycemia, which promotes non-enzymatic glycation of proteins, forming advanced glycation end products, with hemoglobin and albumin being the most clinically relevant targets^[2]^.

Diabetes poses a significant medical and socio-economic challenge, making reliable laboratory markers essential for diagnosis, monitoring, and treatment evaluation. Fasting glucose, the oral glucose tolerance test, and especially glycated hemoglobin (HbA1c) are the current standard methods for diagnosing diabetes. HbA1c offers important advantages over glucose-based tests, although its interpretation may be affected by conditions that alter erythrocyte lifespan, and it does not capture short-term glycemic fluctuations or hypoglycemic episodes^[3–5]^.

Glycated albumin (GA) has emerged as a complementary biomarker for glycemic control. Because of its high abundance and multiple glycation sites, albumin glycation occurs more rapidly than hemoglobin glycation, allowing GA to reflect short-term glycemic status (2–3 weeks)^[2,6–9]^. GA is less influenced by hematologic disorders, such as anemia or hemoglobinopathies, and may provide valuable insights into specific clinical contexts, including renal disease and rapid therapeutic changes^[7–9]^. It has also been linked to the pathogenesis of diabetic complications, particularly in tissues vulnerable to oxidative stress, such as the kidneys^[7]^.

Although diabetes is primarily defined by altered glucose metabolism, it also disrupts lipid homeostasis. Diabetic dyslipidemia is characterized by elevated triglycerides (TG) and reduced high-density lipoprotein cholesterol (HDL-C), reflecting insulin resistance-driven overproduction of very-low-density lipoproteins and accelerated HDL catabolism. Moreover, type 2 diabetes (T2DM) and related metabolic disorders such as prediabetes, metabolic syndrome, obesity, and fatty liver disease are associated with chronic low-grade inflammation, which contributes to both glucose and lipid dysregulation^[10–12]^. GA has emerged not only as an indicator of glycemic status but also as a mediator of inflammatory pathways involved in vascular, renal, and retinal complications^[13]^. At the same time, diabetic dyslipidemia reflects inflammatory processes and further contributes to cardiovascular risk, with qualitative changes in HDL that impair its anti-inflammatory functions^[14–16]^. In this context, evaluating GA together with lipid parameters represents a rational strategy to capture both the glycemic burden and the metabolic-inflammation interplay characteristic of T2DM. Moreover, although evidence remains limited, composite indices combining glycated proteins with lipid parameters, such as elevated triglycerides and reduced HDL-C, may provide a more comprehensive reflection of both glycemic imbalance and lipid dysfunction.

The aims of the present study were to determine GA concentrations in healthy individuals and patients with diabetes, to assess common laboratory parameters of glycemic and lipid status, to compare the discriminatory performance of GA and HbA1c, and to evaluate the discriminatory performance of indices that combine GA and HbA1c with lipid status parameters.

## Materials and methods

### Study population

The present study was carried out in the biochemical laboratories of the Healthcare Institute of the Ministry of Internal Affairs in Belgrade and the Railway Healthcare Institute in Belgrade. Approvals from the Institutional Review Board of both institutions were obtained prior to the initiation of the study (No. 1674/03.11.2023 and 2090/05.06.2024).

The study population consisted of 124 individuals who were referred for blood sampling as part of routine procedure. All subjects provided written informed consent before inclusion in the study. They were divided into two groups: the T2DM group and the control group. Classification into groups was based exclusively on the referral diagnosis: the T2DM group included individuals with a referral diagnosis of type 2 diabetes^[4]^, while the control group consisted of individuals with no referral diagnosis of diabetes, impaired fasting glucose, or glucose intolerance. No additional confirmatory testing (*e.g.*, oral glucose tolerance test) was performed specifically for study purposes.

### Laboratory analyses

Blood samples were collected in the morning, after an overnight fast. After collection, the samples were processed and prepared according to standard laboratory procedures.

The following laboratory tests were performed on the subjects' samples (whole blood and serum, respectively): HbA1c, GA, glucose, total cholesterol, HDL-C, and triglycerides (TG). Glycated albumin was determined by an enzymatic method using a commercially available reagent kit (Instrumentation Laboratory, Milan, Italy) on an ADVIA 1800 biochemical analyzer (Siemens, Munich, Germany)^[17]^. All other analyses were performed by standardized spectrophotometric methods on the ADVIA 1800 and Alinity c (Abbott, Chicago, IL, USA) biochemical analyzers. The ratios HbA1c/HDL-C, GA/HDL-C, HbA1c/TG, and GA/TG were calculated. Low-density lipoprotein cholesterol (LDL-C) was calculated by the Friedewald formula.

### Statistical analysis

Statistical analyses were carried out using MedCalc (version 15.8, Mariakerke, Belgium) and SPSS Statistics (version 26, Chicago, IL, USA) on the Windows platform. Normality of distributions of variables was assessed using the Kolmogorov-Smirnov test. Comparisons of continuous data between groups were performed using the Mann–Whitney *U* test. These data were expressed as median and interquartile range. Categorical data were expressed as absolute and relative frequencies and compared using the chi-square test for contingency tables.

Binary logistic regression analyses were performed to investigate associations between T2DM and glycemic markers and derived indices. Both unadjusted models and models adjusted for sex, age, and BMI were evaluated. The dependent variable was dichotomized: the control group was coded as 0 and the T2DM patients as 1. The independent variables, glycemic markers and indices, were used as continuous variables. The data from the logistic regression analysis were expressed as odds ratio (OR) and 95% confidence interval (CI). The proportion of variance explained by the individual markers was assessed using Nagelkerke *R*^2^.

Receiver operating characteristic (ROC) analyses were performed to evaluate the discriminatory power of individual predictors, glycemic markers, and indices to identify patients with T2DM. The specific cut-offs for GA and HbA1c were determined using Youden's index. Comparisons were also made between the areas under the ROC curves (AUCs). Data from this analysis were presented as AUCs, standard error (SE), and 95% CI. Statistical significance was set at a *P* value < 0.05.

## Results

General characteristics of the study population are shown in ***Table 1***. A higher proportion of men was observed among the patients with T2DM, who were older and exhibited a higher body mass index (BMI) than the controls.

**Table 1:** General characteristics of the study population

Characteristics	Control group (*n* = 63)	T2DM (*n* = 61)	*P*
Age (years)	49 (39–64)	62 (54–69)	< 0.001
BMI (kg/m^2^)	25.4 (23.0–27.0)	27.0 (25.7–30.9)	< 0.001
Male [*n* (%)]	22 (34.9)	40 (65.6)	0.001
Smoking status [*n* (%)]	21 (33.3)	10 (16.4)	0.122
Physical activity [*n* (%)]	23 (36.5)	30 (49.2)	0.251
Continuous data are presented as median and interquartile range, while categorical data are presented as number and percentage. Continuous data were compared using the Mann–Whitney *U* test, and categorical data using the chi-square test.Abbreviations: BMI, body mass index; T2DM, type 2 diabetes mellitus.			

### Biochemical markers and derived indices

Concentrations of biochemical markers are presented in ***Table 2***. Compared with the control group, T2DM patients exhibited significantly higher concentrations of glucose, GA, HbA1c, and triglycerides, while HDL-C levels were significantly lower. No significant differences were observed in total cholesterol and LDL-C between the two groups. Both the HbA1c/HDL-C and GA/HDL-C ratios were significantly higher in patients than in the controls, whereas the HbA1c/TG and GA/TG ratios were significantly lower.

**Table 2:** Biochemical markers of the study population

Parameters	Control group (*n* = 63)	T2DM (*n* = 61)	*P*
Glucose (mmol/L)	5.3 (4.9–5.6)	7.7 (6.8–9.7)	< 0.001
GA (%)	14.4 (13.6–15.2)	17.1 (15.5–19.8)	< 0.001
HbA1c (%)	5.0 (4.8–5.4)	6.4 (6.0–7.5)	< 0.001
Total cholesterol (mmol/L)	5.16 (4.39–5.78)	5.00 (4.29–6.00)	0.386
HDL-C (mmol/L)	1.29 (1.16–1.55)	0.99 (0.90–1.18)	< 0.001
LDL-C (mmol/L)	3.17 (2.58–3.86)	3.02 (2.37–3.52)	0.104
TG (mmol/L)	1.14 (0.77–1.45)	2.00 (1.16–3.00)	< 0.001
GA/HDL-C	11.16 (9.45–12.83)	17.17 (14.11–21.18)	< 0.001
HbA1c/HDL-C	3.92 (3.21–4.50)	6.80 (5.31–7.90)	< 0.001
GA/TG	13.36 (10.28–19.25)	8.46 (5.97–12.67)	< 0.001
HbA1c/TG	4.71 (3.53–6.49)	3.26 (2.36–5.01)	0.002
Data are presented as median and interquartile range and were compared using the Mann–Whitney *U* test.Abbreviations: GA, glycated albumin; HbA1c, glycated hemoglobin; HDL-C, high-density lipoprotein cholesterol; LDL-C, low-density lipoprotein cholesterol; T2DM, type 2 diabetes mellitus; TG, triglycerides.			

### Logistic regression analysis

Univariate logistic regression showed (***Table 3***) that most of the evaluated markers and indices exhibited highly significant associations with the presence of T2DM. The strongest association with T2DM was observed for HbA1c, followed by HbA1c/HDL-C, GA/HDL-C, and GA. Although GA/TG and HbA1c/TG showed statistically significant negative associations with T2DM, their explanatory power was lower (Nagelkerke *R*^2^ = 0.106 and 0.069, respectively) than GA, HbA1c, GA/HDL-C, and HbA1c/HDL-C. Therefore, these indices were not further considered in the subsequent ROC analysis.

**Table 3:** Odds ratios (OR) after univariate and multivariate binary logistic regression analysis for glycemic markers and indices associated with T2DM

Predictors	Unadjusted OR (95% CI)	*P*	Nagelkerke *R*^*2*^	Adjusted OR (95% CI)^a^	Adjusted *P*	Adjusted Nagelkerke *R*^2^
GA (%)	2.388 (1.713–3.329)	< 0.001	0.511	2.538 (1.632–3.947)	< 0.001	0.700
HbA1c (%)	193.000 (26.000–1439.000)	< 0.001	0.801	4519.000 (37.000–548638.000)	< 0.001	0.875
GA/HDL-C	1.510 (1.305–1.746)	< 0.001	0.514	1.545 (1.279–1.865)	< 0.001	0.662
HbA1c/HDL-C	3.121 (2.120–4.595)	< 0.001	0.571	3.336 (1.991–5.588)	< 0.001	0.685
GA/TG	0.909 (0.854–0.967)	0.003	0.106	0.913 (0.844–0.988)	0.024	0.438
HbA1c/TG	0.799 (0.668–0.955)	0.014	0.069	0.797 (0.634–1.003)	0.053	0.425
^ a^Predictors adjusted for sex, age, and BMI. Binary logistic regression analysis was performed.Abbreviations: BMI, body mass index; GA, glycated albumin; HbA1c, glycated hemoglobin; HDL-C, high-density lipoprotein cholesterol; T2DM, type 2 diabetes mellitus; TG, triglycerides.						

When adjusted for sex, age, and BMI, GA, HbA1c, and their composite indices, except for HbA1c/TG, retained significant independent associations with T2DM (***Table 3***). GA, HbA1c, GA/HDL-C, and HbA1c/HDL-C were positively associated with T2DM, while GA/TG showed a negative association with T2DM.

### ROC curve analysis and diagnostic performance

ROC analysis (***Table 4***) demonstrated that HbA1c exhibited the strongest discriminative ability for identifying individuals with T2DM (AUC = 0.966). However, GA also showed very good diagnostic performance (AUC = 0.855). When these areas were compared, the difference between them was significant (area difference: 0.111, *P* < 0.001, SE = 0.020, 95% CI: 0.053–0.169), indicating that HbA1c was the stronger discriminator for T2DM (***Fig. 1***).

**Table 4:** ROC analysis for single markers and indices discriminatory abilities regarding T2DM presence

Predictors	AUC (95% CI)	SE	Sensitivity (%)	Specificity (%)	Cut-off	*P*
GA (%)	0.855 (0.788–0.922)	0.034	80.33	76.19	15.2	< 0.001
HbA1c (%)	0.966 (0.938–0.993)	0.014	91.80	90.48	5.5	< 0.001
GA/HDL-C	0.859 (0.785–0.915)	0.035	72.13	92.06	14.52	< 0.001
HbA1c/HDL-C	0.883 (0.813–0.943)	0.030	85.25	80.95	4.74	< 0.001
Receiver operating characteristic (ROC) curve analysis was performed to assess the discriminatory ability of the markers and indices. Abbreviations: AUC, area under the receiver operating characteristic curve; CI, confidence interval; GA, glycated albumin; HbA1c, glycated hemoglobin; HDL-C, high-density lipoprotein cholesterol; SE, standard error.						

**Figure 1:**
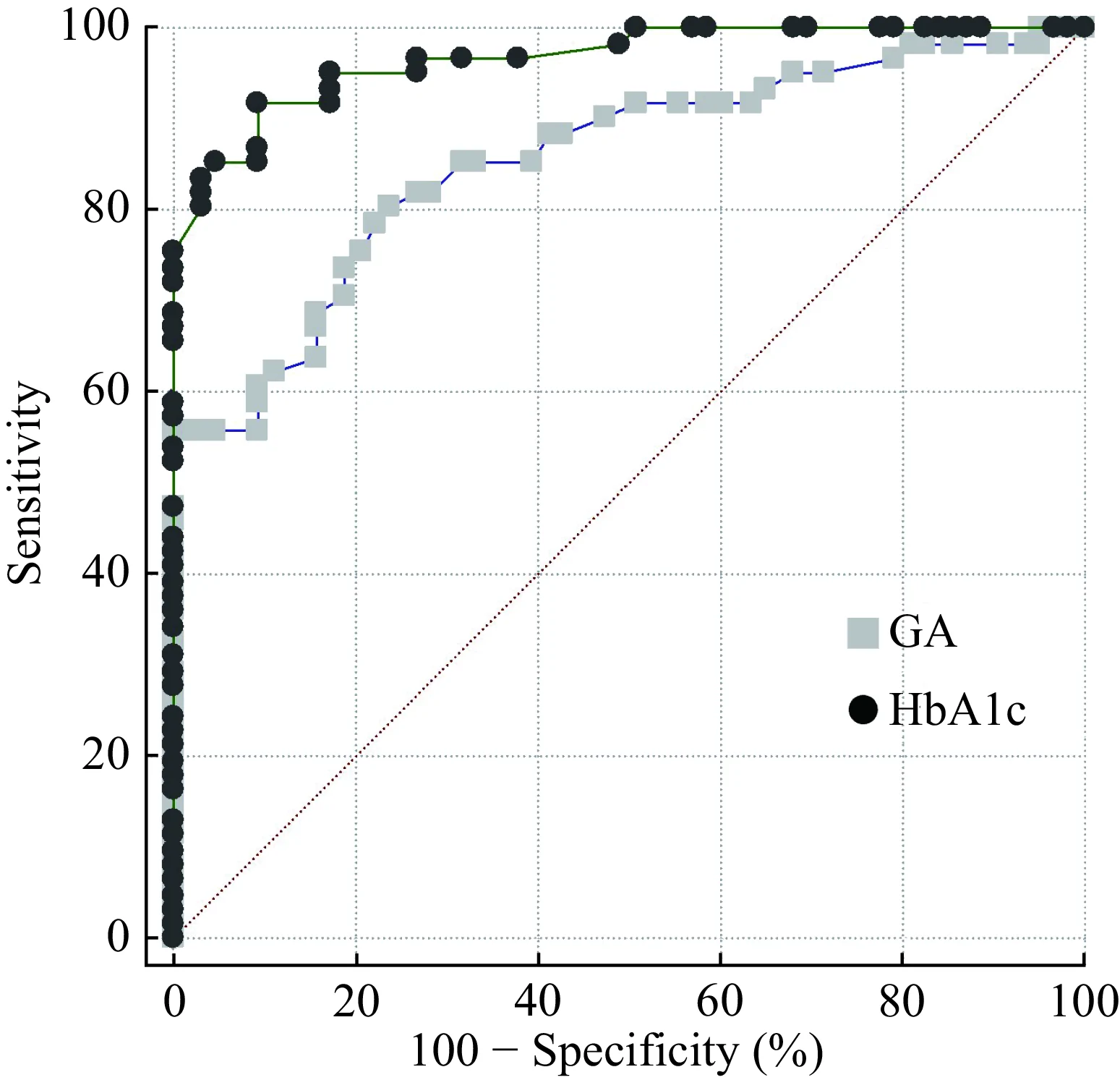
ROC curves for GA and HbA1c in the discrimination of T2DM. The ROC curves illustrate the sensitivity plotted against 100 – specificity (%) for GA (gray squares) and HbA1c (black circles). The diagonal line represents the reference line (AUC = 0.5). Abbreviations: AUC, area under the receiver operating characteristic curve; GA, glycated albumin; HbA1c, glycated hemoglobin; T2DM, type 2 diabetes mellitus.

Although the AUCs for GA/HDL-C and HbA1c/HDL-C were numerically close, their difference (0.0242) was statistically significant (*P* = 0.037, SE = 0.0116, 95% CI: 0.001–0.047); given the small magnitude of this difference, its clinical significance should be interpreted cautiously.

## Discussion

Our study demonstrated the established diagnostic superiority of HbA1c in detecting T2DM, as evidenced by its strong association in logistic regression analysis and highest discriminative performance in ROC analysis. However, GA also emerged as a statistically significant glycemic marker, with an AUC of 0.855, a sensitivity of 80.33%, and a specificity of 76.19%, indicating its clinical relevance. Our findings are consistent with those of the National Health and Nutrition Examination Survey (NHANES) cohort study by Fang *et al*, which reported strong diagnostic utility of GA (AUC 0.824–0.951) in identifying undiagnosed diabetes based on multiple reference definitions^[18]^. Recent longitudinal data from a Korean cohort also support our findings and extend them to a predictive context: this study reported that higher baseline GA levels in healthy individuals were associated with an approximately 2.5-fold increased risk of developing T2DM over a nearly six-year follow-up^[8]^. This reinforces GA's potential role not only as a diagnostic biomarker but also as a prognostic indicator for early diabetes risk. Taken together, our data further substantiate the utility of GA measurements, especially when used in combination with traditional markers, for risk stratification and follow-up monitoring, with early detection remaining a hypothesis for future longitudinal studies.

The exceptionally high odds ratio (OR) values, particularly for HbA1c and type 2 diabetes (OR = 193, 95% CI: 26–1439 in univariate analysis; and OR = 4519, 95% CI: 37–548638 in multivariate analysis), reflect the strong discriminatory power of HbA1c in differentiating between individuals with and without diabetes^[19–20]^, even within our relatively modest sample size. The extremely large OR and wide confidence interval for HbA1c, particularly in the adjusted model, indicate substantial model instability, potentially related to strong separation between groups and the relatively small sample size; therefore, the magnitude of this OR should not be interpreted as a precise effect estimate.

The GA values observed in our study align well with established reference intervals and diagnostic cut-offs reported in the literature. Specifically, the control group exhibited a median GA of 14.4%, consistent with published reference ranges for non-diabetic individuals, typically between 11% and 16%^[21–23]^. The alignment of GA values in our control group with preliminary reference intervals proposed by other authors further supports the robustness of our results and the applicability of GA as a biomarker in non-diabetic populations. The T2DM group demonstrated a significantly higher median GA of 17.1%, which exceeds diagnostic thresholds commonly reported in studies where cut-offs range from 15.5% to 16.0%^[23–24]^, but is in line with data from other sources reporting thresholds between 16.5% and 17.8%^[18,25]^. Clearly, GA values vary depending on population and assay characteristics. It is also worth noting that, to the best of our knowledge and based on available published data, this is the first study to report GA values in a Serbian population. The inclusion of a Serbian cohort extends the available data on GA to a population that has been less well represented in previous studies. The fact that GA levels in our diabetic group substantially exceeded some of the proposed reference ranges underscores GA's discriminative capacity and diagnostic potential in differentiating between normoglycemia and hyperglycemia. These findings reinforce the clinical relevance of GA as a biomarker for diabetes and highlight its sensitivity in detecting glycemic abnormalities, even in a moderately controlled diabetic population. It is also important to acknowledge that recent evidence has pointed to limitations in the standardization of GA assays^[26]^, emphasizing the need for harmonized reference materials and improved cross-platform comparability before GA can be reliably integrated into routine clinical practice.

Although ROC curve comparisons in our study indicate that GA does not outperform HbA1c in terms of diagnostic power, our findings support the validity of GA as a complementary glycemic marker—particularly due to its independence from erythrocyte lifespan and its sensitivity to short-term glycemic fluctuations not captured by HbA1c. These characteristics make GA especially useful in clinical scenarios where HbA1c may be misleading or less reliable, such as in the presence of altered red blood cell turnover or other confounding factors. Patients with specific comorbidities, such as anemia and chronic kidney disease (CKD), represent subgroups in which the diagnostic performance of glycemic markers may differ due to underlying pathophysiological mechanisms. In anemia, the shortened lifespan of erythrocytes and altered hemoglobin turnover can lead to variable HbA1c levels, thereby attenuating its reliability as a marker of average glycemia^[2]^. Similarly, in CKD, multiple factors can compromise the accuracy of HbA1c: uremic toxins, increased carbamylation of hemoglobin, frequent use of erythropoiesis-stimulating agents, and shortened red blood cell lifespan all contribute to a distortion of HbA1c values^[7,27]^. GA is not affected by these mechanisms to the same extent, and this mechanistic distinction supports the potential clinical value of GA as a more reliable diagnostic tool in patient subtypes where HbA1c is compromised.

Regarding lipid profile parameters, our results showed lower HDL-C and higher TG levels in the diabetic group, which are consistent with previously reported and expected findings^[3]^. Reduced HDL-C is a well-documented feature of diabetic dyslipidemia and is linked to insulin resistance, systemic inflammation, and impaired reverse cholesterol transport^[16,28–29]^. Our results are in line with these findings, and HDL-C was significantly lower in individuals with T2DM. Its inclusion in composite indices likely enhances their ability to reflect both glycemic and lipid-related disturbances, offering a more integrated assessment of glycemic-lipid status in T2DM.

Furthermore, the composite indices combining glycemic markers with lipid parameters (GA/HDL-C and HbA1c/HDL-C) demonstrated notable associations with T2DM and yielded good diagnostic performance. The GA/HDL-C index achieved good diagnostic accuracy (AUC = 0.859), closely paralleling that of GA alone, but with a higher specificity (92.06%), suggesting that HDL-C may act as a metabolic modulator within these composite indicators. The inclusion of HDL-C in the ratio may capture additional aspects of metabolic dysfunction beyond glycemia alone, possibly reflecting insulin resistance and lipid metabolism disturbances common in T2DM. Similarly, the HbA1c/HDL-C ratio showed a strong association with T2DM, suggesting that integration of long-term glycemic burden with lipid status may provide complementary metabolic information, although it did not improve overall discrimination compared with HbA1c alone. These findings underscore the role of GA in integrated glycemic-lipid assessment and support the use of GA/HDL-C as a meaningful index in clinical evaluation. Recent studies further highlight that HDL abnormalities in T2DM extend beyond reduced HDL-C levels, involving structural and functional impairments that compromise its anti-atherogenic properties. In agreement with Martagon *et al*, who reported that hyperglycemia alters HDL particle composition and impairs its function in T2DM, our GA/HDL-C and HbA1c/HDL-C indices may reflect these complex interactions, reinforcing their potential as integrated biomarkers of glycemic-lipid status, although their relevance to cardiovascular risk was not directly assessed in this study^[16]^. It is worth noting that although the GA/TG and HbA1c/TG ratios were statistically significant in logistic regression analysis, their explanatory power was low. This may reflect greater biological variability in TG levels^[30]^. Elevated triglycerides in our T2DM cohort likely reflect underlying metabolic and inflammatory disturbances. TG-based markers have been linked to hypertension, cardiovascular disease, hepatosteatosis, and T2DM itself^[10–12]^. This provides a pathophysiological rationale for GA/TG and HbA1c/TG indices, which, despite lower explanatory power due to biological variability in TG, may capture aspects of metabolic inflammation not fully reflected by glycemic markers alone.

The biological rationale for including HDL-C in composite indices lies in the fact that HDL-C is not merely a lipid parameter, but also a marker of reverse cholesterol transport, antioxidant defense, and anti-inflammatory activity—all of which are impaired in T2DM and influenced by glycation^[14–16]^. By combining glycemic markers with HDL-C, the derived indices may better reflect the overall metabolic dysfunction characteristic of diabetes, rather than glycemia alone. Composite indices such as GA/HDL-C and HbA1c/HDL-C provide a synergistic approach by integrating information from two metabolically distinct but pathophysiologically interconnected domains: chronic hyperglycemia and lipid imbalance. While GA and HbA1c capture cumulative glycemic exposure, HDL-C reflects lipid-associated insulin resistance and oxidative stress. This dual-layer approach may increase sensitivity to subtle metabolic derangements that are not captured by single markers. Prior studies have suggested that glycation-related alterations of HDL particles reduce their protective roles in diabetic individuals^[16]^. Because HDL functional properties were not directly measured, whether these composite indices capture qualitative HDL dysfunction remains speculative and warrants further investigation

The practical advantage of composite indices is that they are inexpensive and readily available tools. In particular, the GA/HDL-C and HbA1c/HDL-C ratios may serve as simple, yet powerful indicators of composite glycemic-lipid status, especially in resource-limited settings where advanced panels are not available. It is plausible that GA/HDL-C and HbA1c/HDL-C capture an interaction effect between glycation burden and HDL functionality. Future studies could explore whether these indices correlate with oxidative stress markers or HDL particle remodeling in T2DM. Although the GA/HDL-C and HbA1c/HDL-C indices demonstrated strong associations with T2DM and robust diagnostic accuracy, they should not be viewed as stand-alone alternatives to HbA1c, which remained the most powerful individual marker in our study (AUC = 0.966). Instead, these composite indices are better understood as complementary tools that may enhance diagnostic specificity and offer a more integrated view of metabolic status. The ROC-derived cutoffs were optimized for discrimination within this study population and should not be interpreted as proposed clinical diagnostic thresholds.

A key limitation of our study is the potential underestimation of GA's diagnostic performance due to the relatively well-controlled glycemia in the diabetic group. Most participants with T2DM exhibited only moderately elevated HbA1c, likely reflecting recruitment from primary healthcare settings rather than specialized centers. Although this may have attenuated GA's discriminative capacity, it suggests that GA may retain discriminatory value even among patients with relatively well-controlled T2DM. An additional important limitation concerns the classification of study participants. Because participants were classified according to pre-existing clinical diagnoses of T2DM, the possibility that HbA1c measurements may have contributed to the original clinical classification cannot be excluded. Therefore, the observed diagnostic performance of HbA1c should be interpreted primarily as discrimination between the predefined study groups rather than as an estimate of diagnostic accuracy in an unselected screening population. Demographic differences in age, sex, and BMI existed between the case and control groups. Nevertheless, logistic regression analyses adjusted for these variables demonstrated that GA, HbA1c, and composite indices remained significant predictors, suggesting that these differences did not materially affect the main outcomes. However, the magnitude of the ORs should be interpreted with caution, as they are influenced by both the biological strength of association and the limited sample size. Information on glucose- and lipid-lowering therapies was limited; therefore, treatment-related effects on GA, HbA1c, HDL-C, and TG, and consequently on the derived composite indices, could not be adequately assessed. Data on other comorbidities were also limited, and the study was conducted in only two primary care centers, which may restrict generalizability. These factors should be considered when interpreting the results, and validation in larger, more diverse cohorts is warranted. Future studies should aim to explore relationships between glycemic and lipid markers in larger and more diverse populations, including stratification by renal function, anemia status, and degree of glycemic control.

In conclusion, our study demonstrated that GA concentrations were significantly higher in T2DM patients than in healthy individuals, exceeding commonly reported diagnostic thresholds and supporting GA's relevance as a marker of glycemic status. While it may not replace HbA1c as the primary diagnostic tool, our results demonstrated that GA also provided good discriminative ability and emerged as a statistically significant glycemic marker, highlighting its clinical utility. Composite indices combining GA or HbA1c with HDL-C demonstrated strong associations with T2DM; the GA/HDL-C index, in particular, showed particularly high specificity, suggesting its valuable role in integrating glycemic and lipid parameters for a more comprehensive glycemic-lipid assessment, although these indices did not improve overall discrimination compared with HbA1c alone. In summary, these findings support the complementary role of GA and GA-based indices alongside traditional markers in distinguishing individuals with established T2DM from controls, and underscore their potential additional value in individualized glycemic-lipid assessment, with early disease detection remaining a hypothesis to be tested in future longitudinal studies. Future research should explore the use of GA-based indices in longitudinal studies, and assess their utility not only in discriminating disease status but also in monitoring therapeutic response and predicting diabetes-related complications.
